# Supplementary material for: ‘People like you?’: how people with hypertension make sense of future cardiovascular risk—a qualitative study
Source: BMJ Open. 2018 Nov 8;8(11):e023726. doi: 10.1136/bmjopen-2018-023726 (PMC6231586; doi:10.1136/bmjopen-2018-023726)
Supplement: Supplementary data [file bmjopen-2018-023726supp001.pdf]

Semi-structured interview topic guide for interviewer

Introduction (5 minutes)

- Thank you for taking part
- Check the participant has received and read the information leaflet
- Discuss the interview: lasts about 45 minutes, covers experiences with blood pressure, future health, and information – the topics shouldn't be intrusive or distressing.
- Let me know if you want to take a break, or there are any questions you prefer not to answer.
- Mention that the interview is confidential. If they agree their own GP will be told they took part, but not about what they said.
- Mention that the interviewer has no access to medical records
- Ask if any questions
- If happy to proceed, ask to sign consent form.

Participant introduction (5 minutes)

- Tell me a bit about yourself
  - Age
  - Occupation
  - Country of origin, and self-described ethnicity
  - How long living in the area
  - General health (check about high blood pressure, how long since diagnosed, and if on any treatment)

Main interview (35 minutes)

Future health

- What effects do you think that high blood pressure could have?
  - [Further probe]: short and long term effects, and good and bad effects? Effects in the past? Possible effects in future?
  - [Further probe - if stroke/cardiovascular disease not mentioned by participant]: Have you heard about a link between high blood pressure, and stroke or heart attacks?
- [Taking each effect identified by the participant in turn]: how likely do you feel this is to happen to you?
  - [Further probe if needed]: do you feel these are certain to happen? Unlikely? Likely? How likely or unlikely?

Treatment for high blood pressure

- Do you have a regular treatment?
- How and when do you take your treatment?
  - [Further probes]: Have there ever times that you forget? Are there times that you decide not to take? Have there ever been times that you change the dose/number of tablets? Do you use any other treatments – e.g. herbal?

- What effects do you feel that your medication could have?
    - [Further probe]: short and long term effects, and good and bad effects? Effects in the past? Possible effects in future?
    - [Further probe - if stroke/cardiovascular disease not mentioned by participant]: Do you feel that high blood pressure medication has an effect on stroke or heart attacks?
- [Taking each effect identified by the participant in turn]: how likely do you feel this is to happen to you?
    - [Further probe if needed]: do you feel these are certain to happen? Unlikely? Likely? How likely or unlikely?

Information

- Do you remember getting any information when you were diagnosed with high blood pressure?
    - [Further probes]: from your doctor or nurse? Written or spoken information? From others – friends/family? About high blood pressure itself, and about the treatment?
- Did you find this useful? What were the good and bad parts of it? Could it be improved?
- How important is getting information for you?
- What do you feel are the most important things to tell others in future who are considering starting a treatment?

Conclusion

- Thank you for coming, and for taking part
- Would you like us to send you a copy of the study results? [Ask for an email address or postal address if they do.]
